# Rate of antibiotic prescriptions in German outpatient care – are the guidelines followed or are they still exceeded? 

**DOI:** 10.3205/dgkh000310

**Published:** 2018-03-13

**Authors:** Janine Zweigner, Elisabeth Meyer, Petra Gastmeier, Frank Schwab

**Affiliations:** 1 Department of Hospital Hygiene and Infection Control, University Hospital Cologne, Cologne, Germany; 2 Institute for Hygiene and Environmental Medicine, National Reference Centre for the Surveillance of Nosocomial Infections, Charité – University Hospital, Berlin, Berlin, Germany

**Keywords:** outpatient setting, antibiotic prescribing, general practitioner, over-prescribers, infection

## Abstract

**Aim:** The consequences of antibiotic overuse are substantial. We combined and analyzed the infection diagnoses and antibiotic prescribing practices of physicians in outpatient settings. Recommendations for targeting policy efforts to focused areas are given.

**Methods:** Antibiotic prescriptions and infections diagnosed were provided by a German statutory health insurance provider over a 12-month period. Antibiotic use was expressed as prescriptions per 100 patients.

**Results:** 2,594,000 patient-physician contacts within twelve months were analyzed. A median of 6.5 antibiotics was prescribed to 100 patients. Antibiotic use in private practice showed large variations between and within medical specialties (the upper quarter of physicians who prescribed above the 75^th^ percentile of all prescriptions, at a rate of approximately 43%), by season (antibiotic prescription was 50% higher in winter than in summer) and a considerable proportion of the antibiotics prescribed did not conform with the recommendations of national guidelines. Fluoroquinolones, predominantly ciprofloxacin, were among the top three antibiotics prescribed by all physicians (except pediatricians), although national guidelines do not recommend these agents for uncomplicated respiratory or urinary tract infections. Respiratory tract infections headed the list for the prescription of antibiotics.

**Conclusions:** Antibiotics were still not prescribed appropriately in respect to indication and selection (often unnecessary and/or too broad). We recommend focusing on I) high/over-prescribers, because improved and appropriate antibiotic prescription by this group would result in an over-proportionally lower antibiotic prescription rate, II) respiratory tract infections, because they represent the vast majority of infections treated in primary care and III) intelligent implementation strategies of guidelines.

## Zusammenfassung

**Fragestellung:** Übermäßiger und nicht leitliniengerechter Einsatz von Antibiotika in der medizinischen Versorgung von Patienten hat gravierende Auswirkungen, wie die Zunahme von Antibiotikaresistenzen zeigt. In einer Kohortenstudie wurden die Infektionsdiagnosen und Antibiotikaverschreibungen der niedergelassenen Ärzte mit der Zielsetzung kombiniert und analysiert, Strategien zum rationalen Einsatz von Antibiotika abzuleiten.

**Methode:** Alle Antibiotikaverschreibungen und Infektionsdiagnosen, die niedergelassene Ärzte in Brandenburg in einem 12-Monats-Zeitraum bei einer der größten gesetzlichen Krankenkassen abgerechnet haben, wurden erfasst und analysiert. Der Antibiotikaverbrauch wurde als Verschreibung pro 100 Patienten dargestellt.

**Ergebnisse:** Innerhalb von 12 Monaten fanden 2.594.000 Patienten-Arzt Kontakte statt, die analysiert werden konnten. Im Median wurden 6,5 Antibiotika/100 Patienten verschrieben. Das Verschreibungsverhalten variierte stark zwischen aber auch innerhalb der einzelnen Facharztgruppen (ein Viertel der Allgemeinmediziner verschrieb überproportional viel, allein 43% der Gesamtantibiotikaverordnungen) und zwischen den Jahreszeiten (die Antibiotikaverordnungen waren im Winter 50% höher als im Sommer). Die häufigste Indikation für eine Antibiotikatherapie waren Atemweginfektionen.

Ein beträchtlicher Teil der Verordnungen deckte sich nicht mit den nationalen Leitlinien. Fluorchinolone, insbesondere Ciprofloxacin, gehörten zu den 3 häufigsten Antibiotika, die von allen Fachärzten mit Ausnahme der Pädiater verschrieben wurden, obwohl das weder für unkomplizierte Atemweginfektionen noch für Harnweginfektionen in den Leitlinien empfohlen wird.

**Schlussfolgerungen:** Antibiotika wurden in der ambulanten medizinischen Versorgung häufig weder in Bezug auf Indikation noch auf Wirkstoffklasse adäquat eingesetzt (zu viel und/oder zu breit wirksam). Wir empfehlen daher Folgendes: I) den Fokus auf Hochverschreiber zu richten, da hier eine Verbesserung einer indikationsgerechten Antibiotikatherapie überproportional stark den Verbrauch senken dürfte, II) sich auf die Behandlung von Atemwegerkrankungen in der ambulanten Medizin zu konzentrieren und III) die Implementierung von Leitlinien zu verbessern.

## Introduction

The consequences of antibiotic overuse are dramatic, resulting in a severe threat to public health in Europe and worldwide. Antibiotics are pivotal in the selection of bacterial resistance and the spread of resistance genes in humans, animals, and the environment. Repeated and improper use of antibiotics is the primary cause of increases in drug-resistant bacteria [1], [2], [3].

Furthermore, antibiotic exposure affects the human microbiome, nutrient storage, and bone-density levels. Antibiotics cause marked short-term disturbances in the human intestinal bacteria, and antibiotic treatment can be followed by incomplete recovery of the microbiota to its initial composition [4]. Metabolic diseases such as obesity and type 2 diabetes have been linked with alterations in the microbial composition and function. For example, young children who are given repeated courses of antibiotics are at a greater risk of becoming obese than children who received fewer drugs [5].

The United States spent 10.7 billion dollars on antibiotics in 2009, two-thirds of which in primary care [6]. In Germany, antibiotics generated 772 million euros in sales in 2012 [7]. It is estimated that at least over half of the antibiotics are inappropriately prescribed [8]. Therefore, antibiotic overuse not only drives bacterial resistance and disrupts patients’ microbiome, but also wastes urgently needed health care funds. 

More than 80% of all antibiotics in Europe are prescribed in primary care [9]. There are excellent antibiotic consumption data for European countries from the European Surveillance of Antimicrobial Consumption (ESAC) project group and likewise for Germany [10], [11], [12]. However, there are no large scale data from Germany combining antibiotic prescriptions and infection diagnoses.

Health insurance is compulsory in Germany and it consists of two general types: statutory health insurance by “sickness funds” (*Krankenkassen*) and private health insurance. Some of the 120 statutory health insurers (for instance AOK) are very large with millions of members while others might just have a few thousand. About 90% of the population is insured by statutory health insurance. The proportion of physicians is around 3.4 per 1,000 inhabitants. In 2013, more than 181,000 were working in hospitals and around 146,000 in the outpatient setting.

We combined and analyzed infection-related diagnoses and antibiotic prescription rates of doctors in private practice. The data were supplied by one of the largest German statutory health insurance providers. Based on variations in antibiotic prescription rates, this study aimed to develop recommendations for targeting policy efforts to focused areas. 

## Methods

### Data

All data were provided by the AOK *Nordost*, which is one of the largest members of statutory health insurance providers in Brandenburg, one of the 16 German federal states. Brandenburg has a population of 2.5 million. 600,000 inhabitants were insured by the AOK *Nordost* in 2009. Outpatient antibiotic prescriptions and diagnoses were pseudo-anonymized and sent for further analysis to the Charité Institute of Hygiene and Environmental Medicine in Berlin. Due to the medical data protection law, it was not possible for the authors to identify patients and/or physicians. 

All outpatient prescriptions of systemic antibiotics (J01) were classified using the Anatomical Therapeutic Chemical (ATC) classification system. The ATC system divides the active substances into different groups according to the organ or system on which they act and their therapeutic, pharmacological and chemical properties. All diagnoses were encoded according to the International Statistical Classification of Diseases and Related Health Problems ICD-10. Patient data for reimbursement are collected quarterly by physicians. As a consequence, data could only be analyzed quarterly (from the 3^rd^ quarter 2009 to the 2^nd^ quarter 2010) and not by each patient-physician contact. Physicians with at least 50 patients per quarter and with at least one prescription and/or diagnosis were included in the analysis. 

### Antibiotic use

The rate of antibiotic use was expressed as prescriptions per 100 patients.

### Statistical analysis

Data were calculated both as a total and stratified according to the medical specialization and quarter. The results are presented descriptively as mean, median, and the 10^th^ and 90^th^ percentile. 

## Results

A total of 2,022 physicians in Brandenburg fulfilled the inclusion criteria. 2,594,000 patient-physician contacts were analyzed. Antibiotics were prescribed to outpatients 186,546 times within twelve months. This yields a median of 6.5 antibiotic prescriptions per 100 patients (interquartile range 1.7–12.9) (Table 1 (Tab. 1)).

Pediatricians prescribed antibiotics most frequently. However, around 70% of the patients seen by pediatricians had encoded the infection according to the ICD-10 classification. In contrast, only 15% of the patients seen by a general practitioner suffered from an infection. Pediatricians prescribed an antibiotic to every fifth patient with an infection, whereas general practitioners prescribed an antibiotic to more than half of their patients with an infection.

Antibiotic prescription rates varied widely between medical specialties (Table 1 (Tab. 1)). 

Antibiotic prescriptions peaked during the winter months and were more than 50% higher than during the summer: 8.6 in the 1^st^ quarter of 2010 versus 5.6 prescriptions per 100 patients in the 3^rd^ quarter of 2009. This trend was led by ear, nose, and throat (ENT) physicians, general practitioners, and pediatricians, whereas prescribing practices among gynecologists, surgeons, and urologists showed no seasonal variability.

Table 2 (Tab. 2) presents the top three prescribed antibiotics by medical specialty. It is of interest that a fluoroquinolone (mostly ciprofloxacin) ranks among the most frequently prescribed antibiotics within all specialties; the only exception was pediatricians. They preferred penicillins and macrolides.

**Figure 1:**
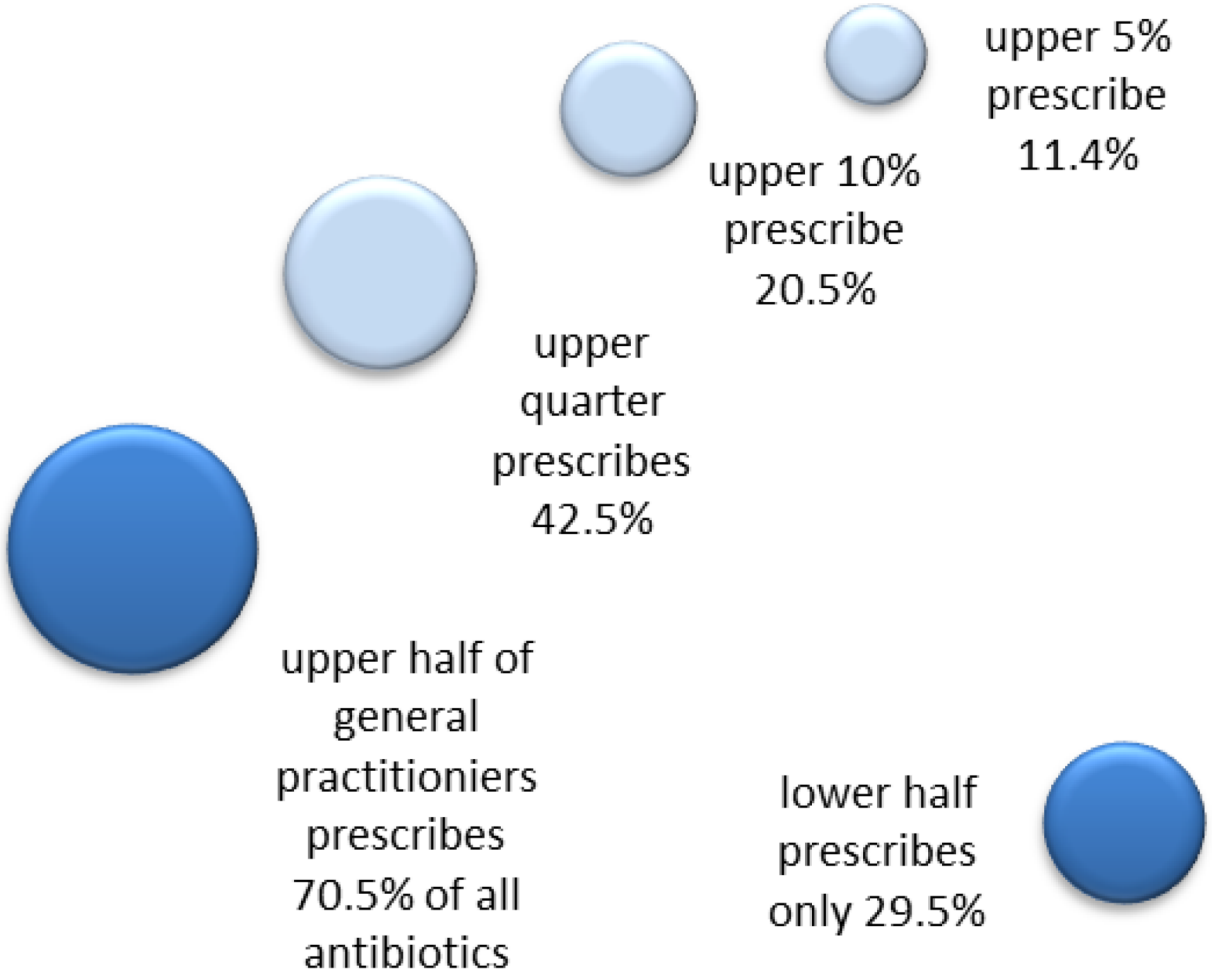
Non-linear antibiotic prescription rate* by general practitioners within 12 months, statutory health insurance (AOK Brandenburg, Germany). *prescriptions per 100 patients

The prescribing of antibiotics is not linear, because a small number of physicians were responsible for prescribing a disproportionally large part of the prescriptions. Figure 1 (Fig. 1) demonstrates this for general practitioners (the largest group of medical specialties in primary care). In the non-linear distribution, the lower half of general practitioners prescribed less than one-third of all antibiotics, whereas the upper half prescribed 71% of all antibiotics. 25% of the high prescribers account for 45% of all antibiotic prescriptions. 

**Figure 2:**
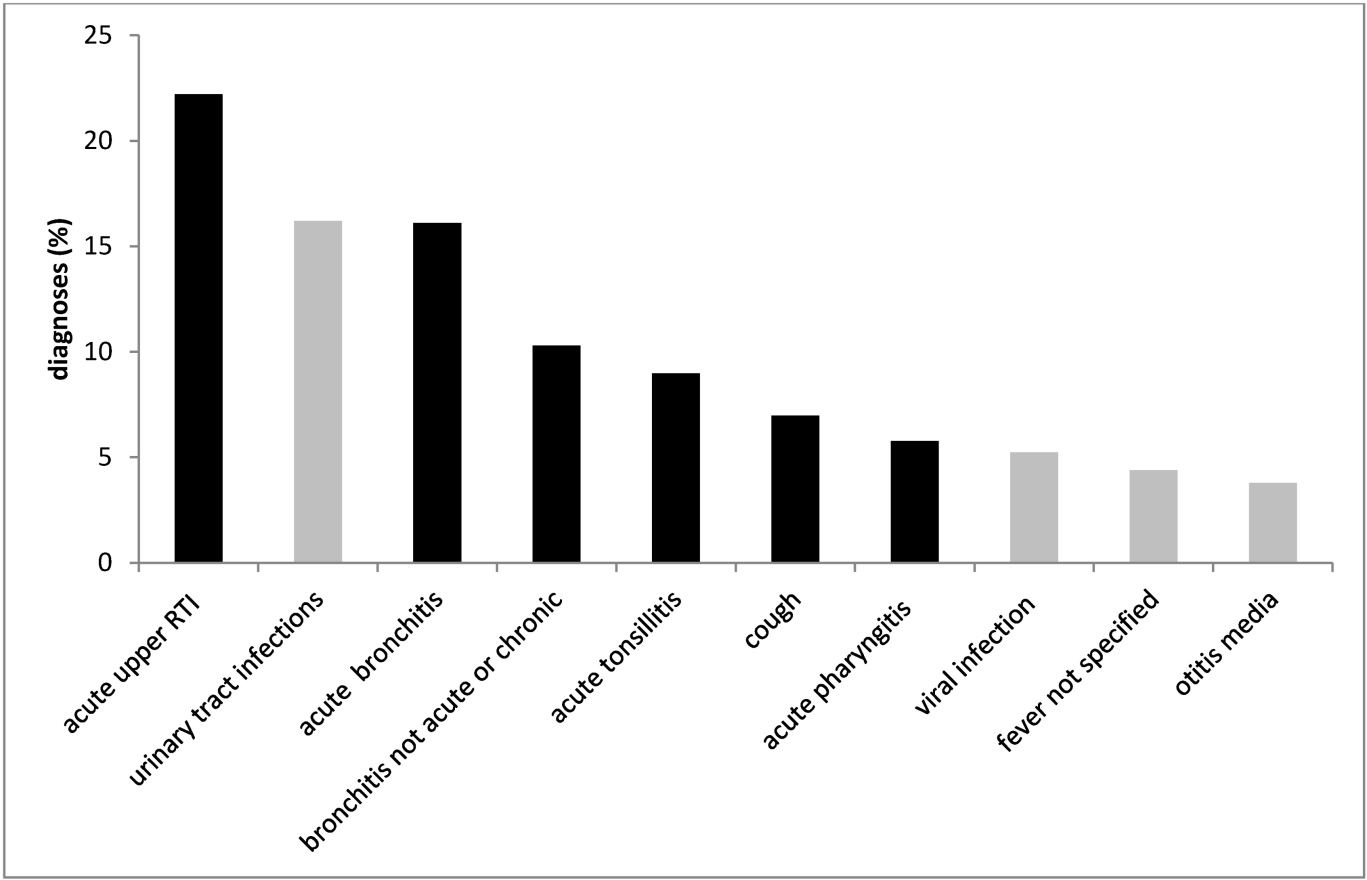
Top ten infections diagnosed in primary care within 12 months, statutory health insurance (AOK *Nordost*, Germany). ICD-10 codes: acute upper respiratory tract infection (J06.9), urinary tract infection (N39.0), acute bronchitis (J20.9), bronchitis not acute or chronic (J40), acute tonsillitis (J03.9), cough (R05), acute pharyngitis (H02.9), viral infection (B34.9), fever not specified (R50.9), otitis media (H66.9). Respiratory tract infections are presented in black columns.

**Figure 3:**
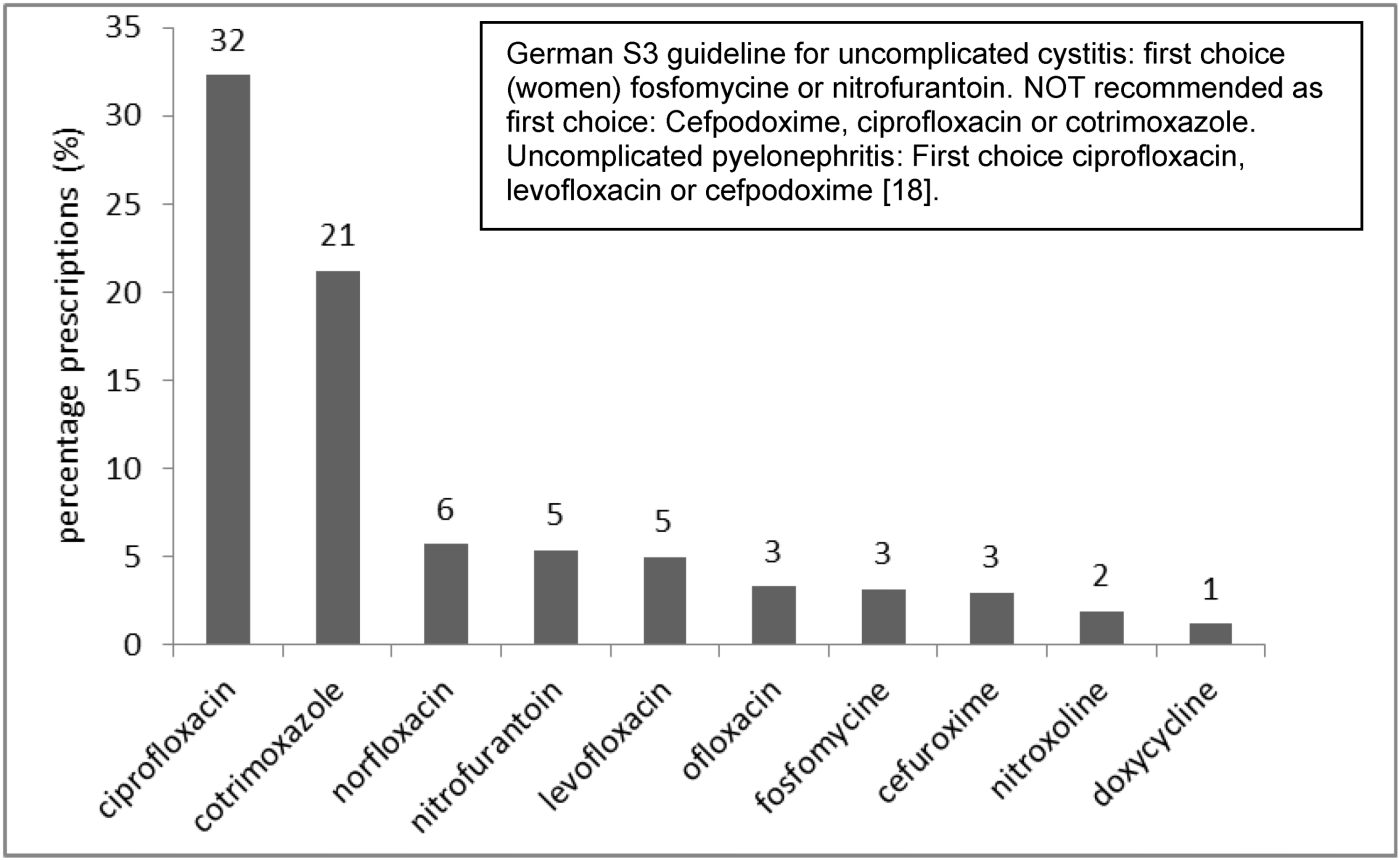
Top ten antibiotics prescribed for urinary tract infections within 12 months, statutory health insurance (AOK *Nordost*, Germany).

**Figure 4:**
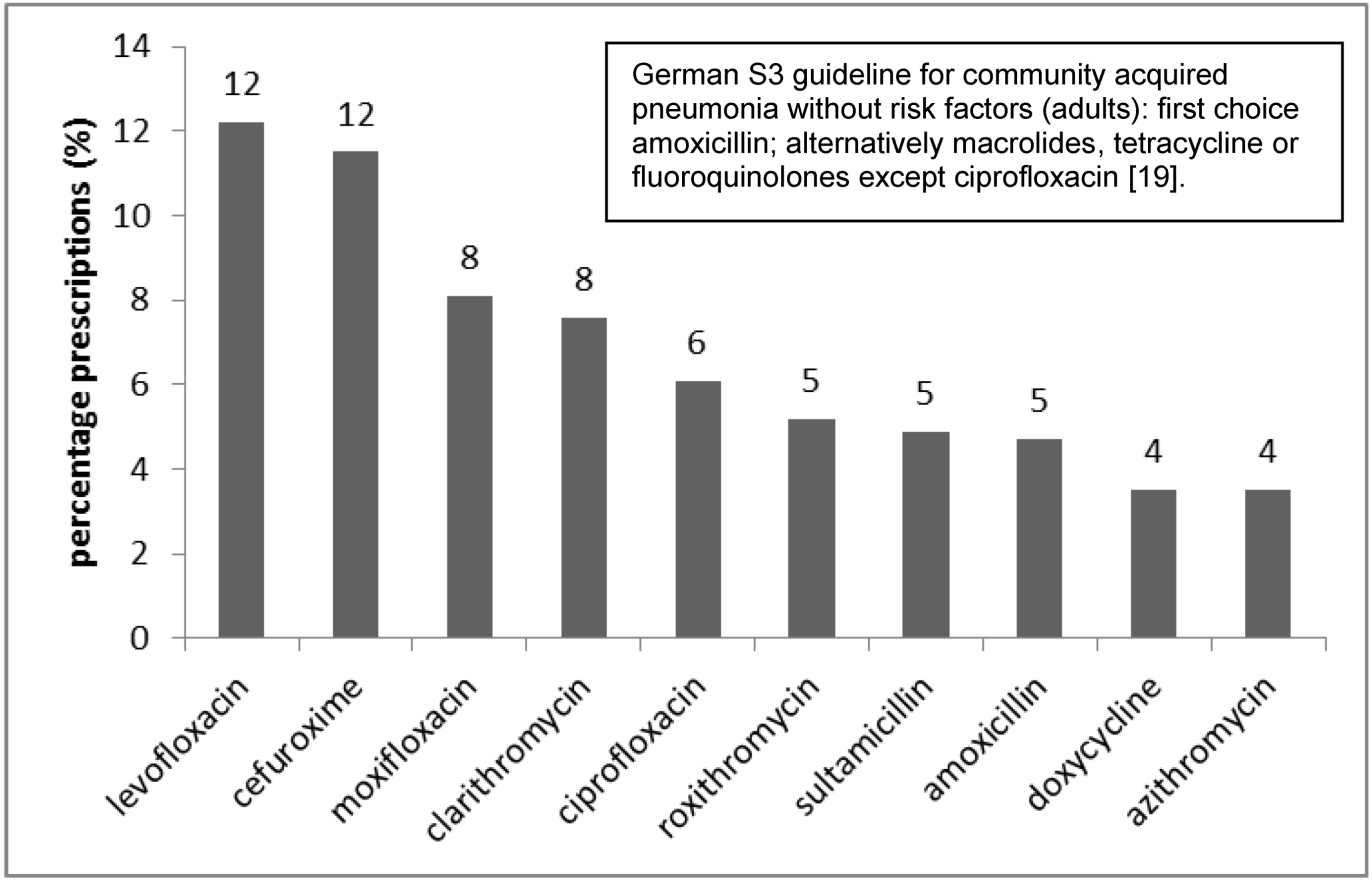
Top ten antibiotics prescribed for respiratory tract infection (for patients without risk factors) within 12 months, statutory health insurance (AOK *Nordost*, Germany).

The top ten infections encoded (ICD-10) and reimbursed by the statutory health insurance company are depicted in Figure 2 (Fig. 2): respiratory and urinary tract infections were the most common. Figure 3 (Fig. 3) shows the antibiotics most often prescribed for urinary tract infections (N39.0) as compared to the recommendation from the German S3 guidelines; the same was done for the treatment of community-acquired pneumonia (Figure 4 (Fig. 4)).

## Discussion

Antibiotic use in primary care showed large variations between and within medical specialties in a non-linear distribution (the upper quarter of physicians prescribed 43% of antibiotics) and by season (antibiotic prescription was 50% higher in winter than in summer), with a considerable proportion of the antibiotics being prescribed inconsistent with recommendations from national guidelines.

The antibiotic use of 7 DDDs per 1,000 insured patients was found to be low in comparison to Europe. This is in accordance with other studies in which Germany ranks in the lower third with an outpatient use density of <15 DDD/1,000 inhabitants – along with the Netherlands, Austria, Scandinavia, Slovenia, Russia, and Switzerland [13]. Significant regional differences also exist within Germany: physicians in the federal states of former West Germany prescribe significantly more antimicrobials than do physicians in the eastern federal states of former East Germany, such as Brandenburg [7], [14]. Furthermore, European and German studies described considerable variations by season. Antibiotic use was much higher in winter than in summer months due to the clustering of respiratory tract infections [7], [13], [15]. It can be hypothesized that antibiotics in winter are largely prescribed for the treatment of upper respiratory tract infections (RTI) although most of these infections are caused by viruses. Therefore, low seasonal fluctuations in antibiotic prescription rates, e.g., in Northern European countries in contrast to Southern Europe, can be used as a quality parameter.

Altiner et al. reported that with an average prescription rate of 50% in German primary care, antibiotics are still too frequently prescribed for RTIs, although it is recognized that antibiotics are very unlikely to alter the course of RTIs, such as throat infections, acute otitis media, maxillary sinusitis, and acute bronchitis [16]. Consequently, current guidelines advise against the use of antibiotics during the initial treatment of uncomplicated RTI in otherwise healthy children and adults. The variation between the medical specialties can be explained by the different number of patients with infections: pediatricians prescribed the most antibiotics but also saw the most patients with infections. Within medical specialties, a large variation was found between low and high over-prescribers that is not explained by case-mix and may be attributed to behavioral factors. The UK government reviewed the literature and stated that many primary care prescribers admit that even some of their own prescribing will not be clinically beneficial. A major factor driving liberal antimicrobial prescribing in primary care is fear of diagnostic uncertainty and its consequences and the perception that their patients will be dissatisfied [17]. A solution for this problem can be rapid point-of-care diagnostics that discriminate between viral and bacterial respiratory tract infections.

Interphysician variability and the choice of antibiotics prescribed reveal problems with appropriate antibiotic prescribing. Broad-spectrum antibiotics such as fluoroquinolones are widely prescribed by all physicians (except pediatricians) although national guidelines do not recommend these agents for uncomplicated urinary tract infections or uncomplicated respiratory tract infections [14], [18], [19]. Increasing resistance rates among uropathogens have complicated the treatment of acute cystitis. Ciprofloxacin resistance of *E. coli* increased in private practice in Germany up to 16% [20].

Taking into account the findings of our study, we would recommend interventions focusing on three points:

1. high/over-prescribers because this would result in over-proportionally lower antibiotic prescription rates
2. RTIs because they represent the vast majority of infections treated with antibiotics
3. the implementation as opposed to mere dissemination of specific guidelines

Key elements of this approach would include the establishment of concrete plans, numbers, and outcome parameters for reduction and prudent use. The Netherlands and their impressive success can be taken as the best-practice example given its more than 50% reduction of (unnecessary) antibiotic use in animals: antibiotic use in food-production animals is reported and feedback is given to veterinarians. According to the benchmarking data, consumption is depicted in red (top 25%), orange, and green zones [21]. Immediate action is mandatory only in the red zones for top prescribers. It seems promising to request action or statements by top prescribers and to offer help. UK researchers showed that social norm feedback from a high-profile messenger can substantially reduce the antibiotic prescription rate at low cost and on a national scale. General practitioners in the feedback intervention group were sent a letter from England’s Chief Medical Officer and a leaflet on antibiotics for use with patients. The letter stated that the physician’s practice was prescribing antibiotics at a higher rate than 80% of the practices in its NHS Local Area Team [22]. This simple intervention resulted in a significant 3.3% reduction of antibiotic items dispensed. In a US-American study among primary care doctors, the use of accountable justification and peer comparison as behavioral interventions resulted in more than 50% lower rates of inappropriate antibiotic prescribing for acute respiratory tract infections [23]. The three interventions were:

- alternatives were presented, e.g., electronic order sets suggesting non-antibiotic treatments
- accountable justification, which prompted clinicians to enter free-text justifications for prescribing antibiotics into patients’ electronic health records
- peer comparison, in which e-mails were sent to clinicians that compared their antibiotic prescribing rates with those of “top performers”

Our study has some limitations. First, it was not obligatory in primary care to encode a diagnosis if antibiotics were prescribed. Therefore, we cannot rule out that some infections were not encoded, although patients suffered from an infection. Second, our data might not be representative because only patients from one statutory health insurance provider from one of 16 federal states in Germany were included.

## Conclusions

Taking these limitations into account, we conclude that a considerable amount of antibiotics in primary care still are prescribed unnecessarily and/or used too broadly, This is lent urgency by the fact that the data from the federal state Brandenburg with the lowest antibiotic prescription density have been analyzed [7], [14]. For reasons of efficacy and efficiency, we recommend targeting the top over-prescribers and cases of antibiotic prescription for respiratory tract infections.

## Abbreviations

- AOK: statutory health insurance by “sickness funds”
- ATC: anatomical therapeutic chemical (classification system)
- DDD: defined daily doses
- ESAC: European Surveillance of Antimicrobial Consumption
- ENT: ear, nose, and throat
- ICD-10: International Statistical Classification of Diseases and Related Health Problems
- RTI: respiratory tract infections

## Notes

### Competing interests

The authors declare that they have no competing interests.

### Ethical approval and consent to participate

The study obtained research authorization and was approved by the institutional ethics committee of the Charité – University Medicine (EA4/118/11).

### Availability of data and materials

The datasets used and/or analyzed during the current study are available from the corresponding author on reasonable request.

### Funding

The study was supported by the German Ministry for Education and Research (BMBF) (IIA5-2509NIK007).

### Authors’ contributions

JZ, EM and PG were responsible for the study design. JZ supervised the study. JZ, EM, PG and FS were responsible for data collection and data cleaning. FS conducted the analysis. All authors interpreted the data, gave important intellectual content, and revised the manuscript critically. All authors read and approved the final manuscript.

### Acknowledgements

We would like to thank Sabine Ebert for excellent support in providing the data from the AOK *Nordost*. 

## Figures and Tables

**Table 1:**
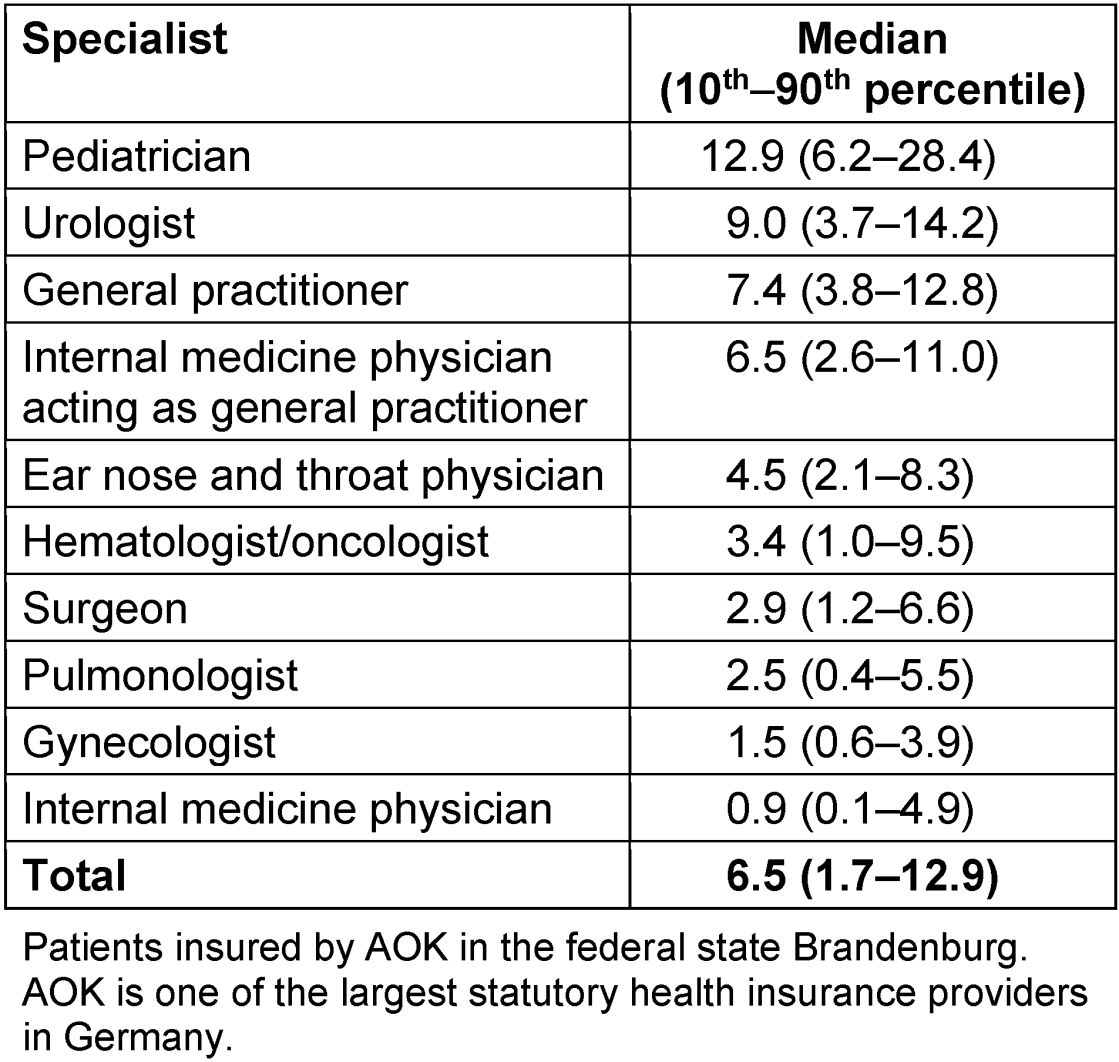
Outpatient antibiotic use per 100 insured patients within 12 months, statutory health insurance (AOK *Nordost*, Germany).

**Table 2:**
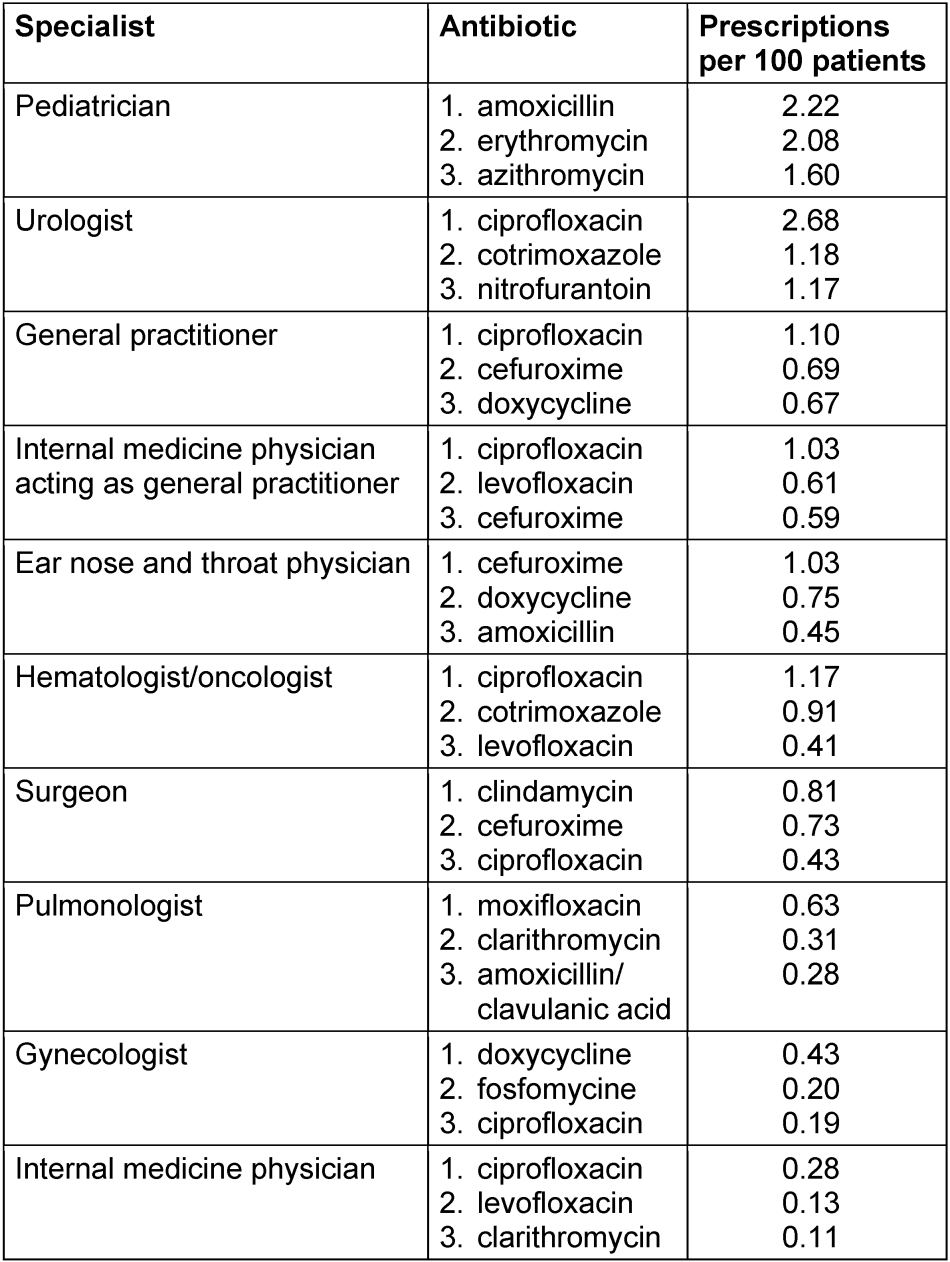
Top three prescribed antibiotic agents by specialty within 12 months, statutory health insurance (AOK *Nordost*, Germany).

## References

[1] Forslund K, Sunagawa S, Kultima JR, Mende DR, Arumugam M, Typas A, Bork P (2013). Country-specific antibiotic use practices impact the human gut resistome. Genome Res.

[2] Zhang Y, Steinman MA, Kaplan CM (2012). Geographic variation in outpatient antibiotic prescribing among older adults. Arch Intern Med.

[3] Costelloe C, Metcalfe C, Lovering A, Mant D, Hay AD (2010). Effect of antibiotic prescribing in primary care on antimicrobial resistance in individual patients: systematic review and meta-analysis. BMJ.

[4] Mikkelsen KH, Frost M, Bahl MI, Licht TR, Jensen US, Rosenberg J, Pedersen O, Hansen T, Rehfeld JF, Holst JJ, Vilsbøll T, Knop FK (2015). Effect of Antibiotics on Gut Microbiota, Gut Hormones and Glucose Metabolism. PLoS ONE.

[5] Bailey LC, Forrest CB, Zhang P, Richards TM, Livshits A, DeRusso PA (2014). Association of antibiotics in infancy with early childhood obesity. JAMA Pediatr.

[6] Suda KJ, Hicks LA, Roberts RM, Hunkler RJ, Danziger LH (2013). A national evaluation of antibiotic expenditures by healthcare setting in the United States, 2009. J Antimicrob Chemother.

[7] Bundesamt für Verbraucherschutz und Lebensmittelsicherheit, Paul-Ehrlich-Gesellschaft für Chemotherapie e.V. (2016). GERMAP 2015 – Bericht über den Antibiotikaverbrauch und die Verbreitung von Antibiotikaresistenzen in der Human- und Veterinärmedizin in Deutschland.

[8] Shapiro DJ, Hicks LA, Pavia AT, Hersh AL (2014). Antibiotic prescribing for adults in ambulatory care in the USA, 2007-09. J Antimicrob Chemother.

[9] Meyer E, Gastmeier P, Deja M, Schwab F (2013). Antibiotic consumption and resistance: data from Europe and Germany. Int J Med Microbiol.

[10] Adriaenssens N, Coenen S, Kroes AC, Versporten A, Vankerckhoven V, Muller A, Blix HS, Goossens H, ESAC Project Group (2011). European Surveillance of Antimicrobial Consumption (ESAC): systemic antiviral use in Europe. J Antimicrob Chemother.

[11] Coenen S, Adriaenssens N, Versporten A, Muller A, Minalu G, Faes C, Vankerckhoven V, Aerts M, Hens N, Molenberghs G, Goossens H, ESAC Project Group (2011). European Surveillance of Antimicrobial Consumption (ESAC): outpatient use of tetracyclines, sulphonamides and trimethoprim, and other antibacterials in Europe (1997-2009). J Antimicrob Chemother.

[12] Augustin J, Mangiapane S, Kern WV (2015). A regional analysis of outpatient antibiotic prescribing in Germany in 2010. Eur J Public Health.

[13] Elseviers MM, Ferech M, Vander Stichele RH, Goossens H, ESAC project group (2007). Antibiotic use in ambulatory care in Europe (ESAC data 1997-2002): trends, regional differences and seasonal fluctuations. Pharmacoepidemiol Drug Saf.

[14] Bätzing-Feigenbaum J, Schulz M, Schulz M, Hering R, Kern WV (2016). Outpatient Antibiotic Prescription. Dtsch Arztebl Int.

[15] Malo S, Bjerrum L, Feja C, Lallana MJ, Abad JM, Rabanaque-Hernández MJ (2014). The quality of outpatient antimicrobial prescribing: a comparison between two areas of northern and southern Europe. Eur J Clin Pharmacol.

[16] Altiner A, Berner R, Diener A, Feldmeier G, Köchling A, Löffler C, Schröder H, Siegel A, Wollny A, Kern WV (2012). Converting habits of antibiotic prescribing for respiratory tract infections in German primary care – the cluster-randomized controlled CHANGE-2 trial. BMC Fam Pract.

[17] Public Health England (2015). Behaviour change and antibiotic prescribing in healthcare settings – Literature review and behavioural analysis.

[18] Deutsche Gesellschaft für Urologie (2017). Interdisziplinäre S3 Leitlinie: Epidemiologie, Diagnostik, Therapie, Prävention und Management unkomplizierter, bakterieller, ambulant erworbener Harnwegsinfektionen bei erwachsenen Patienten. Langversion. AWMF Registriernummer: 043/044.

[19] Ewig S, Höffken G, Kern WV, Rohde G, Flick H, Krause R, Ott S, Bauer T, Dalhoff K, Gatermann S, Kolditz M, Krüger S, Lorenz J, Pletz M, de Roux A, Schaaf B, Schaberg T, Schütte H, Welte T (2016). Behandlung von erwachsenen Patienten mit ambulant erworbener Pneumonie und Prävention - Update 2016. Pneumologie.

[20] Robert Koch Institut Antibiotika-Resistenz-Surveillance. Datenbank Resistenzentwicklung.

[21] Government of the Netherlands Ministry of Economic Affairs (2014). Reduced and Responsible: use of antibiotics in food-producing animals in the Netherlands. Publicatie-nr. EP5_622674.

[22] Hallsworth M, Chadborn T, Sallis A, Sanders M, Berry D, Greaves F, Clements L, Davies SC (2016). Provision of social norm feedback to high prescribers of antibiotics in general practice: a pragmatic national randomised controlled trial. Lancet.

[23] Meeker D, Linder JA, Fox CR, Friedberg MW, Persell SD, Goldstein NJ, Knight TK, Hay JW, Doctor JN (2016). Effect of Behavioral Interventions on Inappropriate Antibiotic Prescribing Among Primary Care Practices: A Randomized Clinical Trial. JAMA.

